# Papain expression in the *Escherichia coli* cytoplasm by T7-promoter engineering and co-expression with human protein disulfide isomerase (PDI) and thiol peroxidase (GPx7) genes

**DOI:** 10.1128/aem.02119-24

**Published:** 2024-11-26

**Authors:** Md Anarul Hoque, Richard A. Gross, Mattheos A. G. Koffas

**Affiliations:** 1 Center for Biotechnology and Interdisciplinary Studies, Rensselaer Polytechnic Institute 8024 , Troy, New York, USA; 2 Department of Chemical and Biological Engineering, Rensselaer Polytechnic Institute 542562 , Troy, New York, USA; 3 Department of Chemistry and Chemical Biology, Rensselaer Polytechnic Institute 428901 , Troy, New York, USA; Danmarks Tekniske Universitet The Novo Nordisk Foundation Center for Biosustainability, Kgs. Lyngby, Denmark

**Keywords:** papain, soluble papain, T7 promoter, *Escherichia coli*, maltose-binding protein, thiol peroxidase, protein disulfide isomerase

## Abstract

Difficulties exist in obtaining full-length, correctly folded, and soluble papain or papain-like proteases that necessitate the exploration of alternative strategies. This study describes the development of an *Escherichia coli* strain capable of producing soluble papain without the need for complex and time-consuming *in vitro* refolding steps. To enhance the production of soluble papain, engineered T7 promoters and a recombinant papain translationally fused with varying tags were constructed. The tags investigated include the maltose-binding protein, small ubiquitin modifier protein, and glutathione transferase. An *E. coli* SHuffle strain was engineered to accumulate hydrogen peroxide (H_2_O_2_) by disruption of the redox pathway. This was accomplished by co-expression of the fusion constructs with two human endoplasmic reticulum-resident proteins, thiol peroxidase glutathione peroxidase-7 (GPx7), and protein disulfide isomerase (PDI). The oxidizing capacity of H_2_O_2_ was used to improve disulfide bond formation in papain. The GPx7-PDI fusion dyad played a significant role in consuming harmful H_2_O_2_ generated by the SHuffle cells. This consumption of H_2_O_2_ helped provide the necessary oxidizing conditions for the efficient production of soluble papain. In shake-flask experiments, the recombinant strain produced ~110 mg/L of papain. Moreover, in batch fermentation, the volumetric yield reached ~349 mg/L. This work provides insights into recombinant papain microbial production that can lead to an industrial viable production strain.

**IMPORTANCE:**

Papain, a cysteine-like protease, has extensive applications across various industries including food, chemical, pharmaceutical, drug, and polymer. However, the traditional isolation of papain from *Carica papaya* plants results in a complex mixture of proteases. Such protease mixtures result in an inability to understand which component enzyme contributed to substrate conversions. Concentrations of constituent enzymes likely differ based on the ripeness of the papaya fruit. Also, constituent enzymes from papaya differ in optimal activity as a function of temperature and pH. Thus, by using papain-like enzymes from papaya fruit, valuable information on component enzyme activity and specificity is lost. Numerous methods have been reported to purify papain and papain-like enzymes from the crude mixture. Often, methods involve at least three steps including column chromatography to separate five cysteine proteases. Such procedures represent tedious processes to manufacture the pure enzymes in *Carica papaya* extracts. The numerous uses of papain for industrial processes, as well as the probability that certain components of papain crude mixtures will be preferred for specific applications, necessitate alternative methods such as recombinant expression from microbial production systems to meet the high world demand for papain.

## INTRODUCTION

Production of papain in prokaryotic cell lines frequently leads to protein aggregation (1, 2). This is a significant challenge in achieving high yields of soluble and functional papain (3). Papain consists of a single polypeptide chain with three disulfide bridges and a sulfhydryl group crucial for its enzymatic activity (4). The formation of native disulfide bonds is not trivial and has evolved in the three cellular compartments in which catalyzed disulfide bond formation commonly occurs, the endoplasmic reticulum (ER) of eukaryotes (5), the intermembrane space of mitochondria (6), and the periplasm of prokaryotes (7). These pathways involve components that catalyze the formation of disulfide bonds. Presumably, the presence of oxygen or small molecules, such as oxidized glutathione, is necessary *in vivo* for disulfide bond formation (8–10). This presumption appeared to explain the fact that proteins with structural disulfide bonds are only found in the more oxidizing noncytosolic intracellular compartments or in the extracellular space (10, 11). Thus, the expression of soluble papain in the cytoplasm of the *Escherichia coli* system is much more challenging due to the lack of an oxidizing compartment. Although inclusion bodies can be produced in high yields (2), their use is problematic as elaborate *in vitro* solubilization, refolding complexities, and purification procedures are required to recover biologically active protein (12, 13). Furthermore, refolding conditions need to be optimized for each protein, and, in many cases, only 15%–25% of inclusion bodies will be converted to bioactive products with loss of activity and yield (14–16). To avoid the complex and time-consuming *in vitro* protein refolding steps required, an alternative strategy for the development of an *E. coli* strain that produces soluble papain is needed.

The multiple factors affect the expression of foreign disulfide bond-containing proteins in *E. coli*. These include promoter strength (17), ribosome binding efficiency (18), the choice of oxidizing compartment periplasm or cytoplasm (19), the presence of helper proteins (20), involvement of eukaryotic ER components (21, 22), and the background of host strains (23). By considering all these factors, the goal of this research focused on developing a suitable expression strain for soluble recombinant papain production in *E. coli*. In the model prokaryotic expression organism *E. coli*, expression of plant cysteine proteases has been limited to the periplasmic compartment (24), mainly due to its native disulfide bond-forming machinery (23). Yet, periplasmic expression requires extensive optimization of conditions (25) to avoid secretion blockage (26) and optimization of targeting signal peptides (27). More importantly, the periplasm lacks ATP (28) and, thus, is devoid of ATP-dependent chaperone systems, resulting in a low capacity for accumulating recombinant proteins. Consequently, the cytoplasm becomes the preferred compartment for recombinant papain expression.

**Fig 1:**
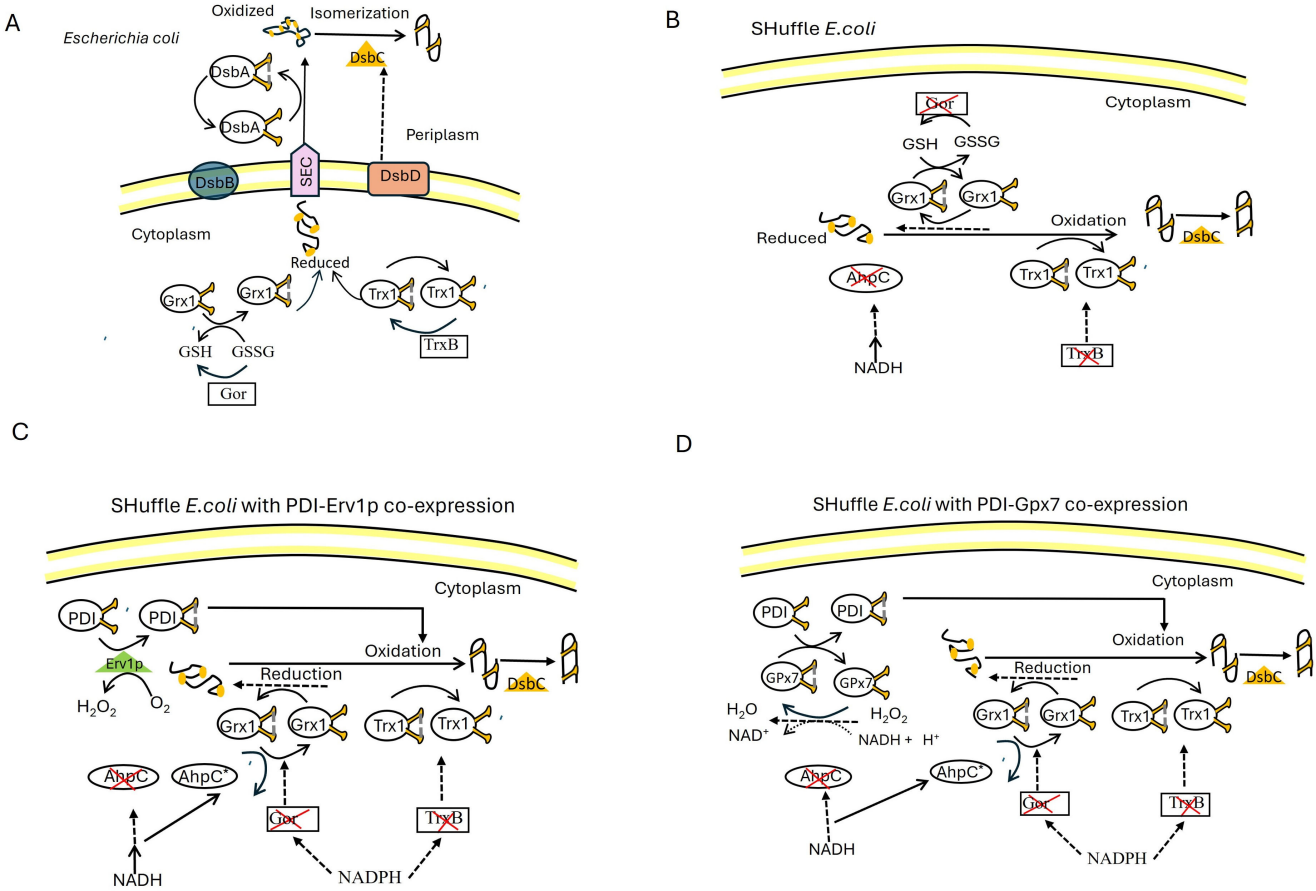
Disulfide bond formation in *Escherichia coli* expression systems. (**A**) The *E. coli* cytoplasm is generally reduced due to high levels of reduced glutathione (GSH) relative to oxidized glutathione (GSSG), maintained by glutathione reductase (gor) and thioredoxin reductase (TrxB). In the periplasm, DsbA facilitates disulfide bond formation. DsbB regenerates DsbA by oxidizing it. Misfolded proteins with incorrect disulfide bonds are processed by DsbC, which requires its redox state to be maintained by DsbD. (**B**) SHuffle *E. coli* has been engineered to create a more oxidizing environment in the cytoplasm. This is achieved by knocking out Gor (reducing the GSH ratio) and TrxB (preventing reduction of Trx1). The reduced ability to maintain a high level of reduced glutathione and the lack of TrxB shift the redox state toward oxidation, allowing disulfide bond formation to occur within the cytoplasm. SHuffle *E. coli* is engineered to cytoplasmically express DsbC, which can isomerize incorrectly disulfide-bonded proteins in the cytoplasm. (**C**) CyDisCo retaining protein disulfide isomerase (PDI)-sulfhydryl oxidase (Erv1P); co-expression in SHuffle *E. coli* further strengthens the oxidation pathway in the cytoplasm. CyDisCo co-expression of PDI readily oxidizes newly translated proteins in the cytoplasm. The redox state of PDI is reset by the sulfhydryl oxidase Erv1p, which generates *de novo* disulfide bonds by donating electrons on to O_2_ and form H_2_O_2_. (**D**) pACYC-duet retaining PDI-glutathione peroxidase-7 (Gpx7) co-expression in SHuffle of the PDI-GPx7 system mediates disulfide transfer from accumulated H_2_O_2_ to recombinant papain via PDI-Gpx7 fusions.

In general, the oxidation of cysteine thiols in cytoplasmic proteins is strongly disfavored for both thermodynamic and kinetic reasons. Second, under physiological conditions, no enzymes can catalyze protein thiol oxidation in the cytosol. The *E. coli* cytoplasm contains two thioredoxins, TrxA and TrxC, and three glutaredoxins (29). The oxidized form of these proteins can catalyze the formation of disulfide bonds in peptides. However, in the *E. coli* cytosol, both the thioredoxins and glutaredoxins are maintained in a reduced state by the action of thioredoxin reductase (TrxB) and glutathione reductase (gor), respectively (Fig. 1A) (30). It has been demonstrated that SHuffle is engineered to address unfavorable redox potential in the cytoplasm to optimize the formation of disulfide bonds in proteins by deletion of trxB and gor along with a suppressor mutation in the peroxidase AhpC (31) . This double ΔtrxB, Δgor deletion is lethal as it abolishes the main reducing pathways responsible for maintaining the redox cycle of the cells (Fig. 1B through D). The lack of peroxidase activity of AhpC accumulated hydrogen peroxide (H_2_O_2_) and caused oxidative stress, which may not only damage the proteome of SHuffle cells but also perturb recombinant protein expression (32).

By considering all these factors, three steps were taken herein to develop an *E. coli* strain for papain production in the *E. coli* cytoplasm. The steps involve screening suitable host strains for cytoplasmic expression, screening suitable fusion proteins regulated by an engineered T7 promoter, and independent co-expression of recombinant pro-papain with fusion proteins containing the ER components protein disulfide isomerase (PDI)-glutathione peroxidase-7 (GPx7) and PDI-sulfhydryl oxidase (Erv1P). GPx7 and Erv1p were found in the endoplasmic reticulum of humans and intermembrane mitochondrial space of *Saccharomyces cerevisiae*, respectively. These two proteins were separately linked with PDI by using a GSG linker and were tested for recombinant papain expression. Gpx7, Erv1P, and PDI are redox-active enzymes involved in the formation of disulfide bonds in the endoplasmic reticulum (Fig. 1C and D) (33). The main difference between these two fusions is that Erv1P can use molecular oxygen and accumulates H_2_O_2_ in the cells (34, 35). In contrast, GPx7 belongs to the peroxidase superfamily and contributes to oxidative protein folding by reducing H_2_O_2_ and donating its disulfide bond to PDI and the protein of interest (Fig. 1D) (32). Oxidized PDI participates in the oxidative folding of proteins both *in vivo* and *in vitro* (36).

In this paper, we describe the expression of papain by using engineered T7 promoters and by constructing a gene network that includes independent co-expression of GPx7-Erv1P or GPx7-PDI in a prokaryotic system for providing a complete oxidizing compartment in the cytoplasm. Co-expression of the human PDI-GPx7 fusion significantly improved the correct assembly and yield of papain, particularly in shake flask (~110 mg/L) and a fermentation processes (~349 mg/L). The developed strain demonstrates an effective route to produce disulfide bond-containing proteins such as recombinant papain and could be used with other papain-like protease expressions. Also, this study provides a road map that, with further improvements in production system titers, can be used for industrial production of pure papain and papain-like enzymes.

## MATERIALS AND METHODS

### Gene cloning, plasmids, and chemicals

The pre-pro-papain cDNA sequence described by Cohen et al. (37) (accession no. M15230) was synthesized by Life Technologies-Invitrogen (Carlsbad, CA, USA) with codon optimization for *E. coli*. The 78-bp (26 amino acids) signal peptide was removed before cloning the pro-papain gene. The ATG start codon was added at the N-terminal of the pro-papain sequence by PCR amplification. Initially, the amplified product was ligated into the expression vectors pET-28a and pET-22b for cytoplasmic and periplasmic expression, respectively. Chromogenic peptide benzyloxycarbonoyl-phenylalanine-arginine-para-nitroanilide (Z-FR-pNa) was purchased from Enzo Life Science (Farmingdale, NY, USA). Protease inhibitor benzamidine HCl, pepstatin, and ethylenediaminetetraacetic acid (EDTA)-free protease inhibitor cocktail were purchased from Thermo Fisher Scientific. Phenylmethylsulfonyl fluoride (PMSF), cysteine-HCl, EDTA, Tris-HCl, and commercial papain were purchased from Sigma-Aldrich.

### Periplasmic expression and purification of pro-papain

The pET-22b-pro-papain plasmid was chemically transformed into *E. coli* BL21 cells. Transformed cells were cultured in 100 mL lysogeny broth (LB) media in shaking flasks. Pro-papain expression was induced by adding 1 mM isopropyl β-D-1-thiogalactopyranoside (IPTG). Induction was performed at an optical density (OD600) of 0.6. The culture incubation was performed at 16°C overnight. After incubation, the cells were harvested by centrifugation. The harvested cells were then resuspended in 50 mL of 100 mM Tris-HCl buffer at pH 8 and centrifuged. Periplasmic extraction was performed using different solutions. The cells were resuspended in 25 mL hypertonic solution (100 mM Tris-HCl, 10 mM EDTA, 20% sucrose, pH 8) and incubated on ice for 10 min. After centrifugation, the supernatant was saved for analysis. The cells were then resuspended in 25 mL hypotonic solution (100 mM Tris-HCl, 10 mM EDTA, pH 8) and incubated on ice for 10 min. After centrifugation (10 min, 16,000 × *g*), the supernatant was analyzed. The pro-papain was purified by following a literature protocol (38).

### Pro-papain cloning under engineered T7 promoters

A previous study by one of us (M.A.G.K.) reported a T7 promoter library prepared by standard site-directed mutagenesis protocols (17). Complementary primer pairs were designed for mutagenesis, and degenerate nucleotides were used to replace the 5-bp T7 RNA polymerase-binding site (17). A 6× His tag was added to the N-terminal of the pro-papain sequence during cloning.

### Screening fusion protein for papain expression

To assess the impact of different fusion proteins on the expression of pro-papain in the *E. coli* cytoplasm, three fusion proteins, namely, maltose-binding protein (MBP), small ubiquitin modifier (SUMO) protein, and glutathione transferase (GST), were considered for evaluation. These fusion proteins were intended to be added at the N-terminus of the pro-papain sequence. The fusion proteins were incorporated into the pETM6-H9 plasmid by using Gibson assembly method. A tobacco etch virus (TEV) cleavage site was included to facilitate the subsequent cleavage of the fusion protein from pro-papain. A His tag was added at the N-terminal of the fusion protein to simplify protein purification.

### Screening suitable *E. coli* as host strain for pro-papain expression

Seventeen commercially available *E. coli* strains were chosen for the screening process. Three redox-engineered strains, specifically Rossetta-gami B, Origami, and T7 SHuffle, were selected for cytoplasmic expression of pro-papain. The remaining strains were selected to potentially identify unknown targets for the optimization of recombinant papain expression. This diverse set of strains includes *E. coli* BL21 (DE3), *E. coli* BLR (DE3), pLyss (DE3), pGro7 (DE3), SoluBL21 (DE3), SoluBL21-pLyss, K4, K5, K92, HB101, W3110, K12, C41, C43, HSM174, MG1655, and SHuffle cells.

### *E. coli* SHuffle cytoplasmic expression and purification of pro-papain

Pro-papain constructs consist of pETM6 plasmid containing consensus T7 promoter and mutated lower-strength promoter H9 and G6. Recombinant plasmids were transformed into Rosetta-gami B cells and T7 SHuffle cells. The transformed cells were cultured on soytone-yeast extract-glycerol (SYEG). The SYEG medium composition included 1.2% soytone, 2.4% yeast extract, 4 mM potassium hydroxide, 2% glycerol, and 25 mM dibasic potassium phosphate, and the pH was adjusted to 7.0 using 4 mM potassium hydroxide. Induction was carried out at 0.5 mM IPTG when the OD600 reached 0.6. The induced cells were incubated in a shaker overnight at 16°C. The cells were then lysed by sonication (10 s × 10 s) using 50 mM Tris buffer with 300 mM NaCl at pH 8.0. The lysis buffer was supplemented with a protease inhibitor cocktail, including 1 mM PMSF, 1 mM benzamidine chloride, 1 mM pepstatin, and 1 mM dithiothreitol. Pro-papain was purified using Ni-NTA affinity chromatography. Elution from the Ni-NTA column was performed with 250 mM imidazole in Tris buffer. Cell pellets were utilized for pro-papain isolation from inclusion bodies following a literature protocol (2).

### Shake-flask culture growth conditions of engineered SHuffle cells

Overnight cultures of 5 mL SYEG media were prepared using appropriate antibiotics. Shake-flask cultures grew at 37°C until the OD600 reached 0.6–0.8. Induction was performed using 0.5 mM IPTG. Post-induction, cultures were further grown at 16°C for 36–40 hours with shaking at 220 rpm. Cells were harvested by centrifugation after the specified incubation period. Harvested cells were lysed by sonication using 50 mM Tris-lysis buffer. The lysate was subjected to purification using a Ni-NTA column. Purified proteins were quantified using a BCA Protein Assay kit (Pierce, cat. no. 23225). The purity of the proteins was assessed by sodium dodecyl sulfate-polyacrylamide gel electrophoresis (SDS-PAGE) and size-exclusion chromatography with multi-angle light scattering (SEC-MALS) analysis.

### Batch fermentation growth conditions

Seed cultures were initiated by transferring a single colony from streaked plates into 250-mL Erlenmeyer flasks containing 125 mL of selective LB liquid medium. Seed cultures were grown at 37°C, shaking at 275 rpm for 14 hours. The grown seed cultures were immediately used to inoculate DASGIP BioBlock fermenters. The fermenters contained 1 L SYEG medium with specific components: 1.2% soytone, 2.4% yeast extract, 4 mM potassium hydroxide, 2% glycerol, 25 mM dibasic potassium phosphate, 0.05% antifoam 204, and selective antibiotics. Fermenters were controlled by a computer running DASGIP software (Eppendorf, Hauppauge, NY, USA). The pH of the culture was maintained at 7 using 28% ammonium hydroxide and 10% phosphoric acid. Cultures were grown at 37°C with an initial agitation speed of 500 rpm. Dissolved oxygen levels were controlled by an agitation/gas flow/oxygen enrichment cascade. IPTG was added when the OD600 reached 0.6, with a final concentration of 0.5 mM in the fermenter. Culture samples were taken regularly (1 mL) to monitor growth rates. After recording the OD600 of the culture, cells were harvested by centrifugation. The collected cell pellet was stored at −20°C. Cells were resuspended into lysis buffer at OD600 = 50. A portion (10 mL) of the resuspended cells was sonicated at 70% maximum amplitude for 10 min (10 s on and 10 s off). Insoluble matter was removed by a 15-min, 13,000-rpm centrifugation. The soluble supernatant fractions containing the expressed protein were collected. Soluble fractions were purified by a Ni-NTA column.

### H_2_O_2_ detection in the engineered SHuffle cells

The co-expression of pro-papain in SHuffle strain with pACYC-Duet-retained PDI-GPx7 fusion and CyDisCo-retained PDI-Erv1P fusion was picked to measure H_2_O_2_ detection. The cell cultures were grown aerobically in SYEG medium and were induced with 0.5 mM IPTG at OD 0.4. The cultures were grown for an additional 2–3 hours at 16°C. Then 1 mL culture for each was centrifuged. The extracellular (supernatant) H_2_O_2_ was measured, with fresh SYEG media serving as control. To measure the intracellular H_2_O_2_, the pelleted cells were resuspended with thymoquinone solution (2 mg/mL) and incubated at 37°C for 30 min. Then thymoquinone treated cells were centrifuged and used for H_2_O_2_ detection, with thymoqto meauinone serving as negative control. The working solution was prepared by following MyQubit Amplex Red (AR) Peroxide Assay protocol (Invitrogen). One hundred fifty-four micrograms of AR was dissolved in 60 µL DMSO, and 60 µL of this solution was then diluted in 6 mL phosphate-buffered saline, pH 7.4, for 100 µM. Then 0.2/mL U of horseradish peroxidase (HRP) was added into this solution. This solution was shielded from light. To measure H_2_O_2_, 100 µL of the sample was mixed with 100 µL working solution in a 96-well plate (black). The reaction continued for 5 min at room temperature and stopped with 40 µL Ultrared stop reagent. Fluorescence was then measured in Spectramax-M5 fluorometer and converted to H_2_O_2_ concentration using a curve obtained from standard samples.

### Papain activation and Z-FR-pNa hydrolysis assay

Pro-papain was activated *in vitro* using 100 mM acetate buffer. The buffer contained 5 mM cysteine-HCl as a reducing agent. Additionally, 2 mM EDTA was included in the activation buffer. The 10 mg/mL enzymes were prepared as stock in 50 mM Tris buffer. The reaction mixture was prepared with different enzyme concentrations: sodium acetate buffer ratio such as 2:1, 1:1, and 1:2, and the activity was measured with chromogenic substrate Z-FR-pNa for papain and other proteolytic enzymes. The reaction mixture of enzyme and buffer was optimized for fusion pro-papain activation and the best enzyme: buffer (1:1) ratio was used to continue experimental work. This study assessed the effect of buffer, incubation time, and temperature for activation. The reaction mixture was incubated for varying time intervals (0, 10, 20, 30, 40, 50, 60, 90, and 120 min). Incubation was carried out at 48°C. The enzymatic activity of the recombinant papain was measured using the chromogenic peptide Z-FR-pNa as a substrate. A 50 µL enzyme solution was incubated with 177 µL sodium phosphate buffer (0.1 M, pH 7.0) and 10 µL Z-FR-pNa (5 mg/mL) at 40°C and 200 rpm. The reaction was stopped after 5 min with 50 µL 50% acetic acid. The reaction mixture was centrifuged, and the hydrolytic release of para-nitroanilide was measured at 40°C at 405 nm. One unit was defined as the amount of enzyme catalyzing the hydrolysis of 1 µmol Z-FR-pNA per minute. All measurements were performed with two biological and two technical controls, ensuring reliability and reproducibility.

### Kinetic parameter analysis

The kinetic measurements were monitored with a spectrophotometer at a constant temperature of 40°C with 1 mL reaction volumes (path length = 1 cm). Kinetic measurements using Z-FR-pNa as the substrate were conducted in phosphate buffer (100 mM, pH 8.0) at concentrations ranging from 0.2 to 2.0 mM. Kinetic measurements using Z-FR-pNa were conducted at concentrations ranging from 0.2 to 2.0 mM. The reaction rates were measured by monitoring the release of *p*-nitroanilide (ε405 = 9,920/M/cm). The background rates of spontaneous hydrolysis in the absence of enzymes were deducted from the enzymatic rates to calculate the actual values. The kinetic parameters were obtained using the Michaelis-Menten equation [*VV* = *S* × *E* × *k*_cat_**/**(*S* + *K*_m_)] with GraphPadPrism software (GraphPad, San Diego, CA, USA), in which *V* is the initial velocity; *E* is the enzyme concentration; *S* is the substrate concentration; *k*_cat_ is the turnover number; and *K*_m_ is the Michaelis-Menten constant.

## RESULTS

### Design recombinant constructs of papain and screening of 17 *E. coli* strains

The prepro-papain cDNA sequence, as described by Cohen et al. (37), consists of 78 bp (26 amino acids) called signal peptide/pre-peptide necessary for ER translocations in eukaryotic systems. This signal peptide is known to be toxic to bacteria and hinders heterologous expression of papain-like cysteine proteases in *E. coli*. For these reasons, it was removed before cloning the pro-papain gene (Fig. S1A). The wild-type codon-optimized pro-papain gene was successfully cloned into the pETM6 vector under the regulation of the T7 promoter for cytoplasmic expression. A 6-unit His tag was added to the N-terminus of the pro-papain sequence to simplify the purification procedure. The His tag containing pro-papain gene was then introduced in the cytoplasm of 17 commercially available *E. coli* strains, and its expression levels in these strains were assessed. Some strains were chosen based on the deletion of *txB*- and *gor*-encoding genes in the host cells, affecting the disulfide bond-reducing power. These strains include Rosetta-gami B, which is a BL21 derivative with seven tRNAs for rare cordons upregulated, including those for glycine and proline and are also deficient in *trxB* and *gor* genes. Origami (DE3), which is a K-12 derivative with mutations in both the *trxB*- and *gor*-encoding genes and is known to greatly enhance disulfide bond formation in the cytoplasm (39). Similarly, SHuffle cells are also deficient in *trxB* and *gor* genes and also carry an additional chromosomally integrated periplasmic disulfide isomerase gene (DsbC), which provides an oxidizing environment for disulfide bond-containing protein expression (40). RossettaGami-B, Origami, and SHuffle diminish disulfide bond-reducing power due to the deletion of the *trxB* and *gor* genes. Other strains were screened, but no improvement was seen in pro-papain production.

**Fig 2:**
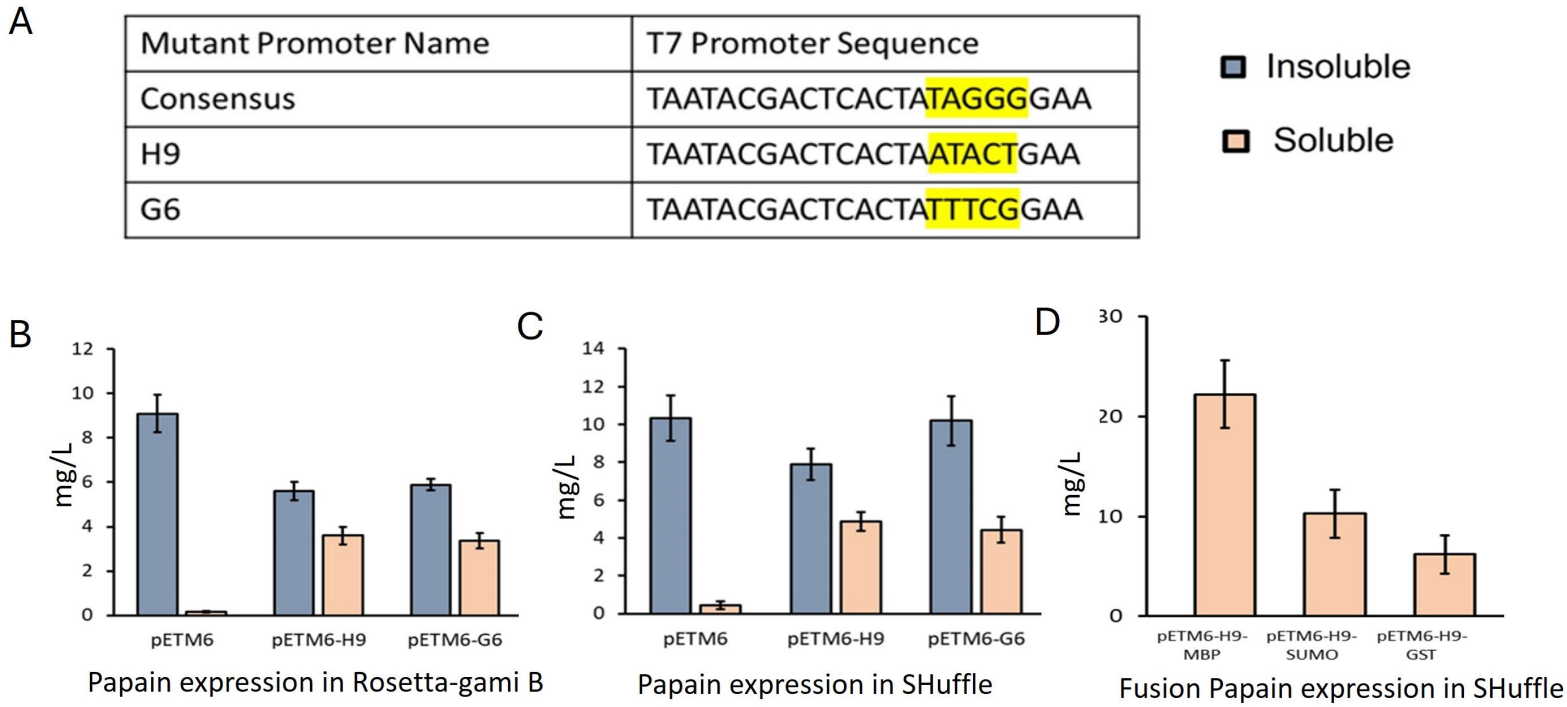
T7 promoter engineering for papain expression. (A) Sequences of mutant T7 promoters from high to low strength. (**B**) Papain expression in Rossetta-gami B. (**C**) Papain expression in T7 SHuffle *E. coli* cells. (**D**) Pro-papain expression with the fusion protein MBP, SUMO, and GST using the mutated H9 promoter in SHuffle *E. coli* cells.

By expressing papain with these strains, we found Rosetta-gami B, Origami, and SHuffle only produced 0.2–0.45 mg/L in the cytoplasm soluble fraction (Fig. S2). None of the other *E. coli* host strains tested produced a soluble papain. These results demonstrated that the redox pathway-engineered *E. coli* host cell is the most essential for soluble recombinant papain production. To determine the expression level of protein aggregates, the isolation of pro-papain from inclusion bodies in cell pellets was carried out. We obtained 8–10 mg/L of pro-papain in inclusion bodies from Rosetta-gami B and SHuffle cells (Fig. 2B and C).

### T7 promoter engineering for papain expression

Next, we considered the possibility that *E. coli* papain production strains may be experiencing metabolic burden due to an overloading of cells with extrinsic mRNAs, reducing the translation of essential intrinsic mRNAs. One potential solution would be to lower the promoter-binding strength to support proper folding and reduce protein aggregation. In a previous study, we developed a T7 promoter library with varying binding strengths to T7 RNA polymerase. To identify the effect of the T7 promoter and corresponding mutated promoters on papain production, a pro-papain encoding gene was cloned in the pETM6, pETM6-H9, and pETM6-G6 plasmids. The background of these three plasmids is identical except that a mutated T7 promoter with lower binding strength compared to the wild-type T7 promoter is present in plasmids pETM6-H9 and pETM6-G6 (Fig. 2A). Expression of these three plasmids into the Rosetta-gami B, RossettaGami-B revealed that pETM6, pETM6-H9, and pETM6-G6 produced 0.21, 3.7, and 3.4 mg/L of pro-papain, respectively (Fig. 2B). To understand the improvement in papain production resulting from reduced binding strength of T7 promoters, the growth rates of Rosetta-gami B, RossettaGami B cells carrying pETM6-pro-papain, pETM6-H9-pro-papain, and pETM6-G6-pro-papain were measured. All the T7-promoter papain expressing strains exhibited slower growth rates than the H9 and G6 promoters (Fig. S4). The probable explanation is a metabolic burden associated with the high translation of papain under the regulation by the wild-type T7 promoter. Expression of pro-papain by different mutated T7 promoters in SHuffle cells resulted in pro-papain titers of 4 to 4.8 mg/L (Fig. 2C). The presence of DsbC enhances the fidelity of disulfide bond formation and yields up to 1.3-fold higher papain than Rosetta-gami B cells. Additionally, lysed cells from both Rosetta-gami B and SHuffle cells were used to purify papain from inclusion bodies by following the protocol developed by Choudhury et al. (2). We obtained 8–10 mg/L of denatured protein from the lysed cell pellets (Fig. 2B and C).

### Screening fusion tags for improved recombinant pro-papain expression in *E. coli* SHuffle

Although lower-binding-strength T7 promoters played an essential role in papain solubility in *E. coli*, the corresponding soluble yields remained low and produced significant fractions of insoluble papain. The insoluble expression of pro-papain in *E. coli* cytoplasm represents a major bottleneck in protein production. To address the solubility issue, we fused pro-papain with large fusion proteins MBP, SUMO, and GST separately because the immunogenicity of the tags and their effect on the structure and function of recombinant proteins are major limitations compared with the use of small fusion tags, and each protein of interest may respond uniquely to the fusion partner (41). A TEV cleavage site (ENLYFQG) was inserted between the fusion partner and pro-papain sequence. Additionally, a 6-unit His tag was added at the N-terminal of the fusion partner to facilitate purification procedures. The validity of the constructed fusion proteins was confirmed through DNA sequencing of the respective transformed recombinant plasmids. Results indicate that MBP, SUMO, and GST fusion partners resulted in approximately 6.0-fold, 2.5-fold, and no significant increase in soluble recombinant papain expression, respectively, compared to the control, where no translational fusion was used (Fig. 2D).

### Co-expression of double plasmid in shuffle for papain expression

**Fig 3:**
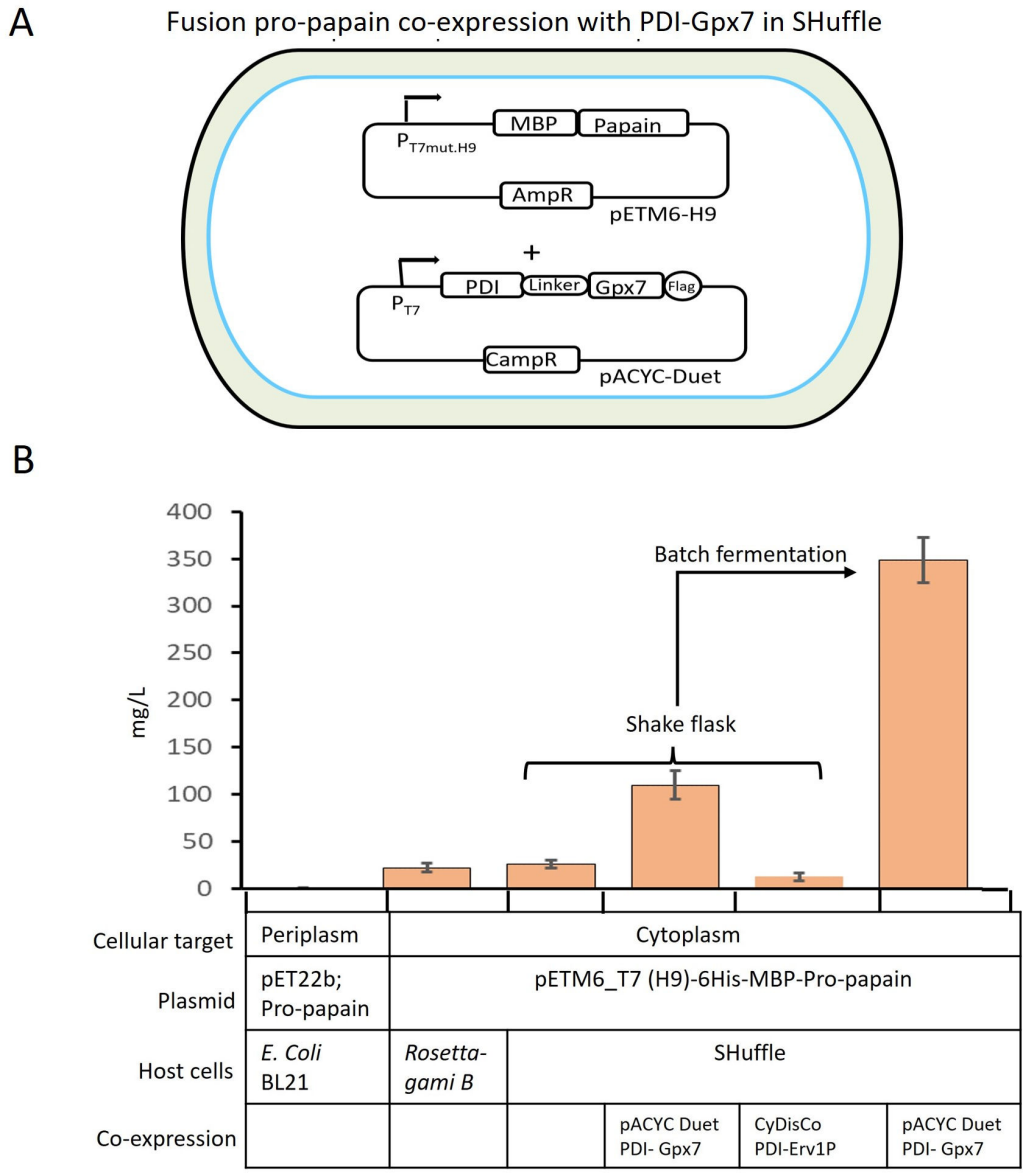
Schematic representation of co-expression system in T7 SHuffle cells. (A) Plasmids; pETM6-H9 vector was used for cloning MBP-pro-papain fusion under the regulation of a mutated T7 promoter (H9) with an N-terminal His tag, and pACYC Duet vector expressing the human PDI-GPx7 fusions. PDI and GPx7 peroxidases were fused by a 17-amino acid linker and cloned under the regulation of a T7 promoter with a C-terminal 3× Flag tag. (B) Papain expression pattern in the shake flask and batch fermentation with different host cells. Periplasmic expression in *E. coli* BL21 by utilizing recombinant pET22b vector, cytoplasmic expression of pro-papain with the pETM6_T7(H9) vector in RossettaGami-B, and co-expression of pro-papain with PDI-GPx7 and PDI-Erv1P in T7 SHuffle cells at 16°C. Bar represents papain expression pattern with different host cells, retaining different cellular target, plasmid, and co-expression of gene pair redox mutations (bottom).

The SHuffle cells contain the deletion of genes encoding trxB and gor, which creates a redox-impaired environment (40). Additionally, a periplasmic copy of DsbC is inserted into the SHuffle chromosome to further enhance disulfide bond formation fidelity (40), resulting in the final Δ*trxB*, Δ*gor*, Δ*ahpC* + c*DsbC* strain, named SHuffle (40). The lack of peroxidase activity of AhpC induces oxidative stress (42), presumably due to the increased amounts of H_2_O_2_. Papain-like cysteine protease is known to be expressed in the endoplasmic reticulum in papaya plants and relies on oxidizing environment for proper folding (43). In eukaryotes, it has been demonstrated that the endoplasmic reticulum-resident enzymes might be responsible for disulfide bond formation (33). This assumption led us to the development of a gene network in the *E. coli* cytoplasm to promote the folding of recombinant papain. In particular, we used the protein disulfide isomerase-thiolperoxidase (PDI-GPx7) from the human endoplasmic reticulum. The fusion of PDI to GPx7 is hypothesized to increase the probability of a H_2_O_2_→GPx7→PDI→papain redox relay pathway. The engineered SHuffle contained two plasmids. The first plasmid, pETM6-H9, contains pro-papain fused with MBP under the regulation of the mutated low-binding-strength T7 promoter H9 (Fig. 3A). A 6-unit His tag was added at the N-terminal of MBP to simplify protein purification. The second plasmid, pACYC-Duet, retained two endoplasmic reticulum-resident proteins: thiol peroxidase GPx7 and PDI from humans. Previous work demonstrated that the PDI-linker-GPx7 fusion allows the active sites of both PDI and GPx7 to potentially face each other (32). The codon-optimized PDI-GPx7 (PDI, NP_000909) and GPx7 (GPx7, NP_001139309.1) were fused by a 17-amino-acid linker, GSGSGSGSGSGSSGSGS (44), along with a C-terminal Flag tag, and cloned into the pACYC-Duet plasmid under regulation of the strong T7 promoter. Expression results demonstrate that the co-expression of PDI-GPx7 fusions in SHuffle strain resulted in the production of 110 mg/L of papain, which is fivefold higher than when expressing only pETM6-H9-pro-papain in the SHuffle cells. Furthermore, a 14-fold increase resulted from performing cultivations in a batch fermentation (Fig. 2B). Similarly, instead of the PDI-GPx7 fusion, the CyDisCo plasmid (45), containing PDI-Erv1P under T7 promoter, was co-expressed with pETM6-H9 plasmid in the SHuffle system. The Erv1p from the intermembrane mitochondrial space of *Saccharomyces cerevisiae* catalyzes the reaction: dithiol + O_2_ → disulfide + H_2_O_2_. The co-expression of pro-papain with the PDI-Erv1P fusion reduced protein production by fourfold than the parental strain (SHuffle) (Fig. 2B). One of the possible causes is that Erv1P increased the H_2_O_2_ level in the cells, which may perturb the recombinant papain production in SHuffle cells. To quantify the H_2_O_2_ level in the SHuffle cells with PDI-GPx7 fusions and PDI-Erv1P fusion, H_2_O_2_ was measured extracellularly and intracellularly by using AR-HRP assay. No measurable H_2_O_2_ was observed extracellularly in either fusion type. On the other hand, the SHuffle cells with PDI-GPx7 fusion and PDI-Erv1P fusion accumulated 0.042 and 0.25 µM H_2_O_2_ intracellularly, respectively. H_2_O_2_ accumulation increased around six times in SHuffle when we co-expressed PDI-Erv1P fusion. The higher intracellular H_2_O_2_ levels in SHuffle cells with PDI-Erv1P fusion compared to PDI-GPx7 fusion are likely due to the combined effects of Erv1P’s sulfhydryl oxidase activity and the absence of thiol peroxidase (aphC). This could indeed impact cell health and recombinant protein yields.

### Optimization of expression parameters, SDS-PAGE, and Western blotting analysis

**Fig 4:**
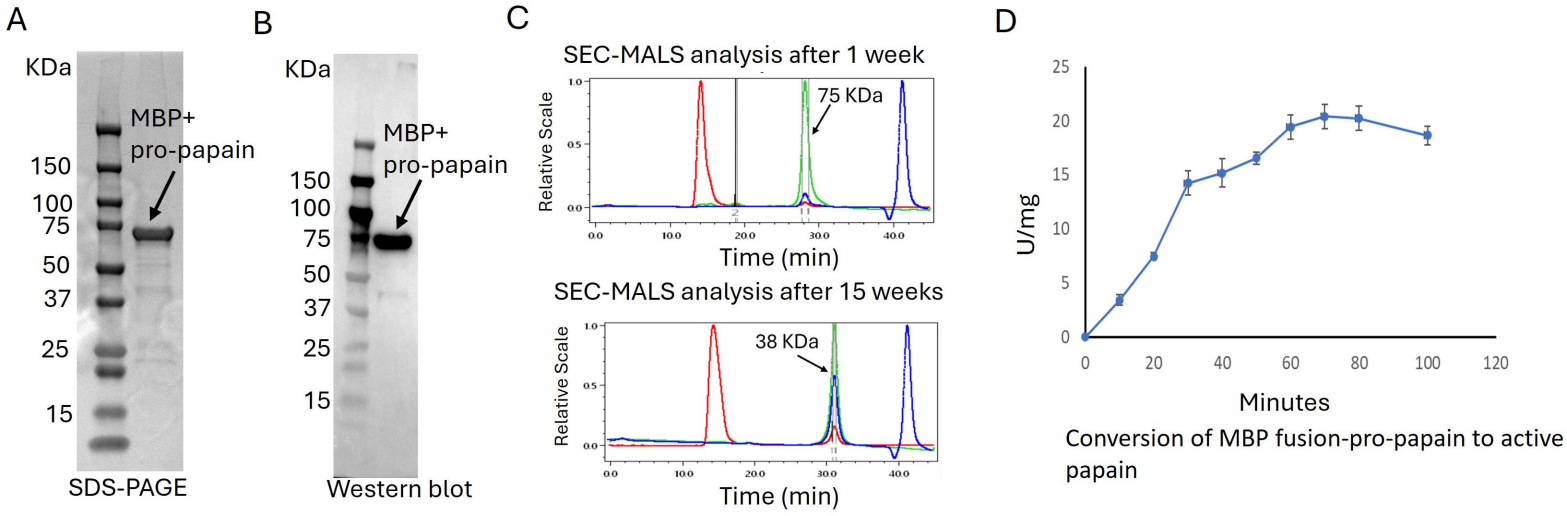
SDS-PAGE analysis of expression and purification of recombinant pro-papain from *E. coli*. (**A**) SDS-PAGE of recombinant pro-papain with a fusion protein (MBP). (**B**) Western blot of MBP-pro-papain. (**C**) SEC-MALS analysis after 1 week (2 mg/mL, injected volume 40 µL) and after 15 weeks (1 mg/mL, injected volume 40 µL). Colors in panel C differentiate from the detector used: green UV, blue refractive index, and red-light scattering. (**D**) Monitoring the conversion of MBP fusion papain to mature papain by determining the evolution of activity as a function of incubation time using as substrate benzyloxycarbonyl-Phe-Arg-pNA.

The expression of recombinant pro-papain with an MBP fusion protein was optimized in SHuffle cells. Experiments were performed at induction temperatures varying from 16°C to 30°C at 0.5 mM IPTG concentration and at 16°C with between 0.1 and 1.0 mM IPTG. The highest papain yield was achieved with 0.5 mM IPTG concentration during a 24-hour cultivation at 16°C in shake flasks (Fig. S3A and B). The highest yield of papain was achieved with 0.5 mM IPTG concentration at 16°C for 24 hours in shake flasks (Fig. S3A and B). Expressed cells were lysed by sonication, and the recombinant protein was purified using Ni-NTA chromatography. The purity of the protein was confirmed by SDS-PAGE (Fig. 4A). Western blot analysis using an anti-His antibody confirmed the presence of a distinct single band at 75 KDa, corresponding to the pro-papain tagged with histidine (Fig. 4B). SEC-MALS analysis was performed to determine protein aggregation and confirm the molecular weight of the MBP-pro-papain fusion. Results after 1-week storage at −80°C showed the presence of the fusion protein, consistent with SDS-PAGE results. However, after 15 weeks of storage at −80°C, the fusion protein (MBP) separated from pro-papain (Fig. 4C). One possible explanation is that the TEV cleavage site is susceptible to the papain protease. Despite the separation observed in SEC-MALS after prolonged storage, the protein activity was not reduced. To address the separation issue during storage, the recombinant pro-papain was lyophilized and stored at −80°C for up to 3 months. Results indicated that the protein activity remained unchanged under these storage conditions.

### Activation of fusion pro-papain to mature papain

The recombinant fusion pro-papain was produced and purified as inactive precursor or zymogen to prevent unwanted protein degradation. Zymogen conversion to the active enzyme typically involves removal of an N-terminal extension of the mature enzyme. The mechanisms of conversion to active enzymes are diverse in nature, ranging from enzymatic to nonenzymatic or simply lowering pH. The optimal conditions for *in vitro* activation of the T7 SHuffle expressed fusion pro-papain to mature papain were determined by using 100 mM acetate buffer, pH 4.0. The reaction mixture was prepared with different enzymes concentrations: sodium acetate buffer ratio such as 2:1, 1:1, and 1:2, and the activity of Z-FR-pNa was measured. Among them, we found the 1:1 ratio as the best enzyme and buffer mixer for the papain activation (data not shown). The effect of buffer and incubation time on activation at 48°C was assessed (Fig. 4D). Hence, SEC-MALS analysis demonstrated that MBP tends to separate from pro-papain and, because their molecular sizes are same, it is hard to determine whether pro-papain is still present. Thus, to assess activation of pro-papain to mature papain, the enzymatic activity of the mature papain was measured using the chromogenic peptide Z-FR-pNa as a substrate as a function of time (Fig. 4D). It appears from activity measurements that maximum conversion of MBP fusion pro-papain to papain occurred between 70 and 80 min.

Comparison of pro-papain activation to activation of the MBP fusion pro-papain under the same incubation conditions and using Z-FR-pNa as substrate was studied using SDS-PAGE. As seen in Fig. S6, the mature papain appeared as a single band by 30 min, suggesting that activation occurred more rapidly for pro-papain. (Fig. S6).

### Papain activation and kinetic analysis

The specific activity of recombinant papain and commercial papain was determined using benzyloxycarbonyl-Phe-Arg-pNA as a substrate at 40°C in phosphate buffer at pH 8.0. The specific activity for recombinant papain and commercial papain (a mixture of papain and corresponding isozymes) was 20.4 ± 0.68 and 14.7 ± 0.56 U/mg, respectively. The catalytic efficiency, *k*_cat_/*K*_m_, of purified MBP-fusion papain, was determined by using chromogenic substrate benzyloxycarbonoyl-phenylalanine-arginine-para-nitroanilide at 40°C in phosphate buffer at pH 8.0, is 2.52 × 10^5^ M^−1^ S^−1^. To assess whether MBP affects the activity we measured the recombinant papain activity obtained by pro-papain activation and found the activity *k*_cat_/*K*_m_ is 2.61 × 10^5^ M^−1^ S^−1^. This result suggested that the presence of MBP fusion does not hamper catalytic efficiency.

## DISCUSSION

In this study, we aimed to enhance the efficiency of disulfide bond formation in pro-papain and localized it to the cytoplasm of *E. coli*. This was achieved by altering the cytoplasmic environment through genetic modifications, leading to improved solubility and biological activity of recombinant proteins. To achieve this, two synthetic plasmids were introduced into the cytoplasm of SHuffle cells to alter disulfide bond formation and enhance solubility. The first plasmid, pETM6-H9, contained pro-papain with MBP under the regulation of a mutated T7 promoter (H9). The second plasmid, pACYC-Duet, carried the two endoplasmic reticulum-resident proteins thiol peroxidase GPx7 and PDI. As an alternative, CyDisCo retained PDI-Erv1P fusion. An initial attempt involved expressing papain in the *E. coli* periplasm, but low (µg/L) expression levels were observed. Later, 17 *E. coli* strains were screened for cytoplasmic expression, 3 of which (Rosetta-gami B, Origami, and T7 SHuffle cells) showed promise. These three strains have mutations in *trxB*- and *gor*-encoding genes, impacting disulfide bond formation. However, only SHuffle cells had the periplasmic isomerase DsbC in the cytoplasm, facilitating correct folding. To reduce protein aggregation and formation of inclusion bodies, a mutated T7 promoter with lower binding strength was employed. Introduction of reduced strength T7 promoters significantly increased protein production levels in SHuffle cells. Furthermore, fusion partners, such as MBP, SUMO, and GST, were investigated for their impact on papain folding and solubility. MBP fusion with papain under the mutated T7 promoter H9 increased yields in SHuffle cells (Fig. 2). Two co-expression systems were explored to mimic the eukaryotic redox potential in the cytoplasm. Co-expressing PDI-Erv1P fusion protein results in six times higher accumulation of H_2_O_2_ compared to the PDI-GPx7 fusion. One of the possible reasons is that the SHuffle strain lacks alkyl thiol peroxidase (Aph−), leading to higher levels of this reactive oxygen species. Another reason is that PDI-Erv1P fusion contains Erv1P, which is a sulfhydryl oxidase, that facilitates the transfer of electrons from the thiol groups in proteins to oxygen, producing H_2_O_2_ as a by-product. The elevated H_2_O_2_ levels can be toxic and may inhibit cell growth. In contrast, effective management of oxidative stress through PDI-GPx7 fusion system allows cell health and improved protein production in shake flasks and batch fermentation, indicating a redox cascade involving H_2_O_2_→GPx7→PDI→papain. Consequently, decreasing the proximity between GPx7 and PDI would allow for H_2_O_2_ oxidized GPx7 to oxidize PDI, recapitulating the PDI-GPx7 redox cycle in its native ER compartment. Future work will explore whether further improvements could be achieved by supplying minimum acceptable levels of exogenous H_2_O_2_ or engineering a triple fusion of PDI-GPx7-Erv1P to enhance H_2_O_2_ supply to GPx7.

In conclusion, this work represents a successful effort in designing a system that promotes efficient and soluble expression of pro-papain in the cytoplasm of *E. coli*. The engineered T7 promoter and the co-expression strategy, involving the introduction of human GPx7-PDI fusion protein, demonstrates improvements in the correct assembly of pro-papain as well as fermentation production titer (349 mg/L), where previously no soluble fractions of pro-papain were reported in *E. coli* cytoplasmic expression. Although engineering an artificial gene circuit often requires trial-and-error attempts and are not optimized by the selective processes of evolution, the functionality of this artificial system could be used for recombinant papain or papain-like cysteine protease production from different sources.

## Data Availability

All data generated during this study are included in this paper and the supplemental material files.

## SUPPLEMENTAL MATERIAL

Supplemental material aem.02119-24-s0001.docx Figures S1 to S7; Table S1.

ASM does not own the copyrights to Supplemental Material that may be linked to, or accessed through, an article. The authors have granted ASM a non-exclusive, world-wide license to publish the Supplemental Material files. Please contact the corresponding author directly for reuse.
