# Supplementary material for: Papain expression in the Escherichia coli cytoplasm by T7-promoter engineering and co-expression with human protein disulfide isomerase (PDI) and thiol peroxidase (GPx7) genes
Source: Appl Environ Microbiol. 2024 Nov 26;91(1):e02119-24. doi: 10.1128/aem.02119-24 (PMC11784408; doi:10.1128/aem.02119-24)
Supplement: Supplemental material — Figures S1 to S7; Table S1. [file aem.02119-24-s0001.docx]

**Supporting Information**

Papain Expression in the *Escherichia coli* Cytoplasm by T7-promoter Engineering and Co-Expression with Human Protein Disulfide Isomerase (PDI) and Thiol Peroxidase (GPx7) Genes

Md Anarul Hoque^a^^,c^, Richard A Gross,^a,b^ Mattheos A. G. Koffas^a,c^#

^a^Center for Biotechnology and Interdisciplinary Studies, Rensselaer Polytechnic Institute, Troy, NY 12180, United States

^b^ Department of Chemistry and Chemical Biology, Troy, New York, USA

^c^ Department of Chemical and Biological Engineering, Troy, New York, USA

Running Head: Papain Expression in the *Escherichia coli*

#Address correspondence to Mattheos A. G. Koffas, koffam@rpi.edu

KEYWORDS: Papain, Soluble papain, T7 promoter, *Escherichia coli*, Maltose binding protein, Thiol peroxidase, Protein disulfide isomerase.


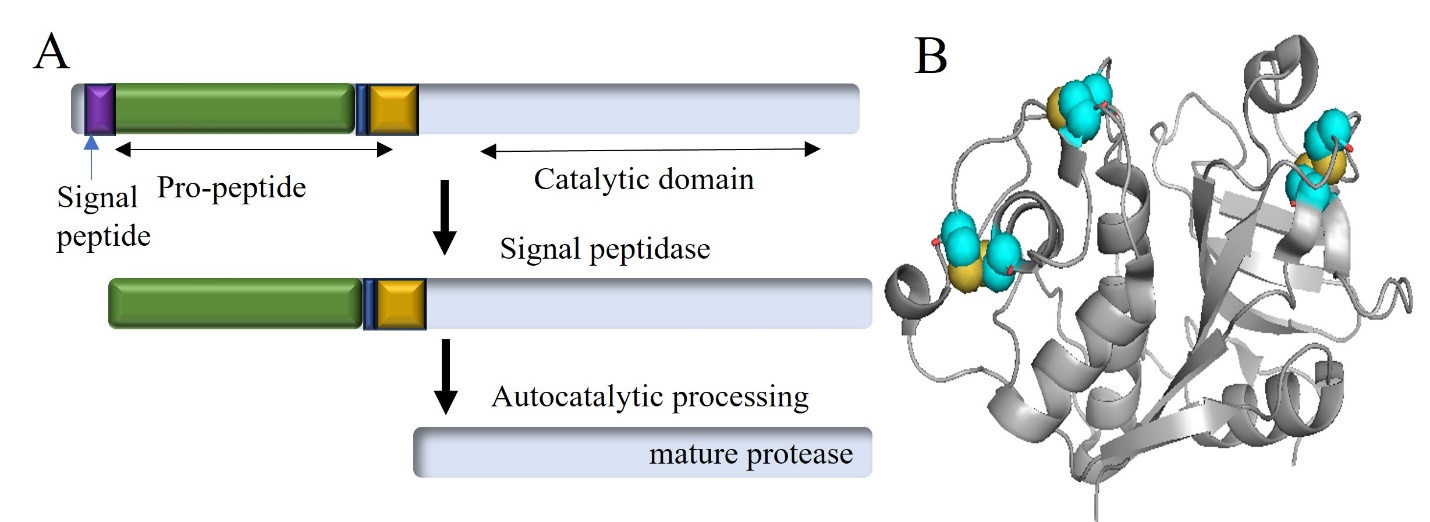


**FIG S1** A) Three domains of the papain are signal peptide (26 aa), pro-peptide (115 aa), and catalytic domain (212 aa) B) Papain structure (pdb:1ppn) and three disulfide bonds are shown in sphere.


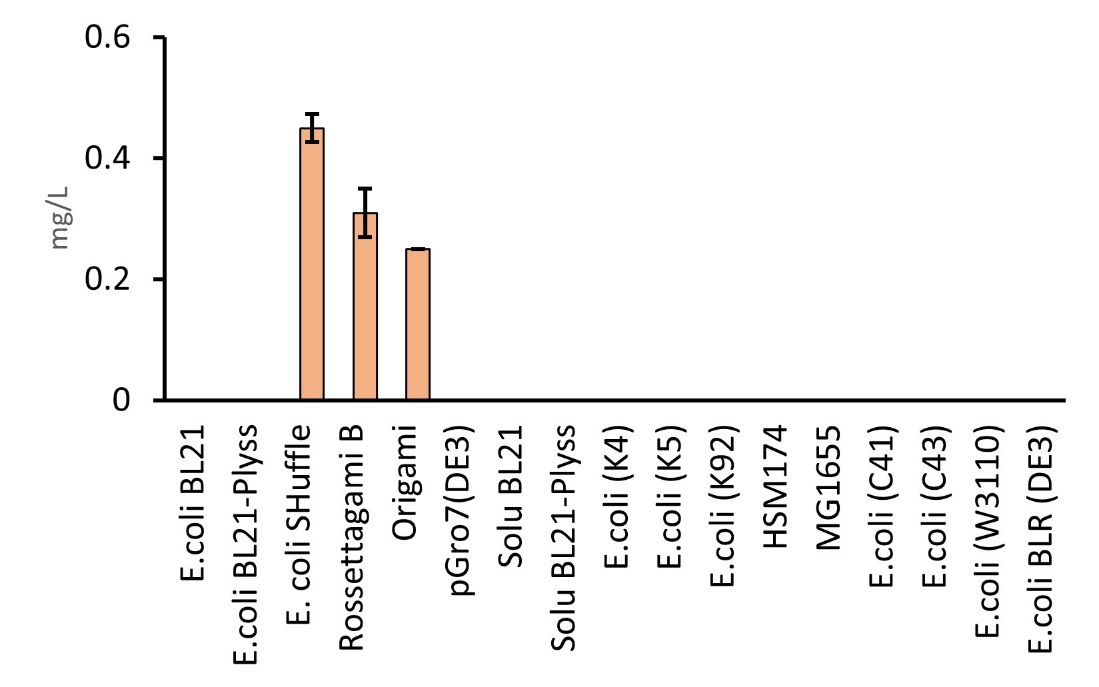


**FIG S2** Cytoplasmic Expression of Papain in Different Host Cells


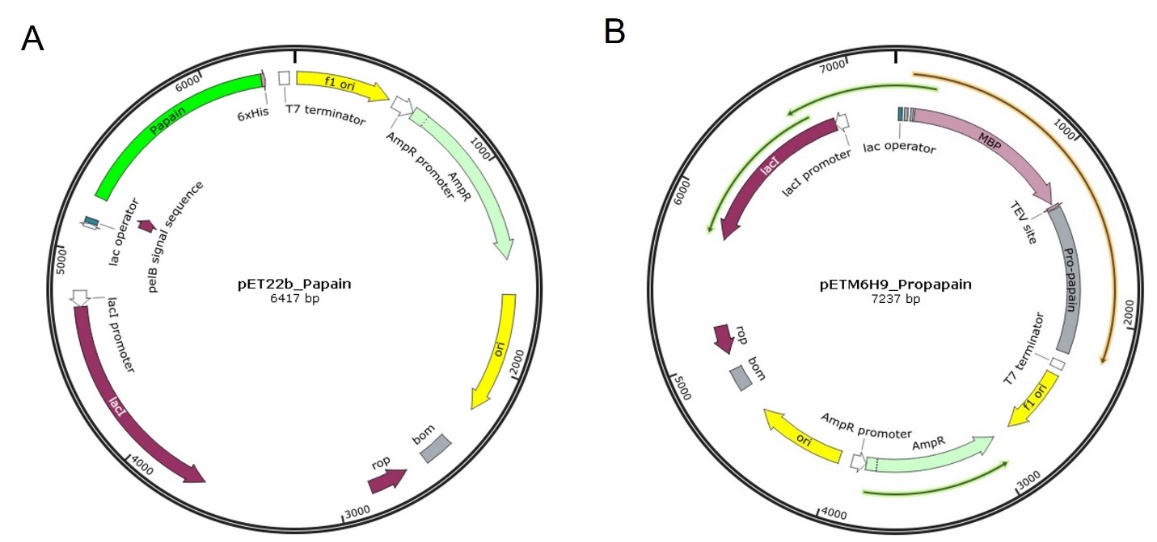


**FIG S3** Cloning pro-papain (A) plasmid map of pET22b-pro-papain, where pro-papain sequence was fused with pelB signal sequence for periplasmic expression (B) plasmid map of pETM6H9-pro-papain. In this plasmid MBP was fused with pro-papain under T7 mutated promoter H9.

**FIG S4** Growth curve of *Rossettagami-B* in LB media for pETM6-papain, pETM6-H9-papain, and pETM6-G6.


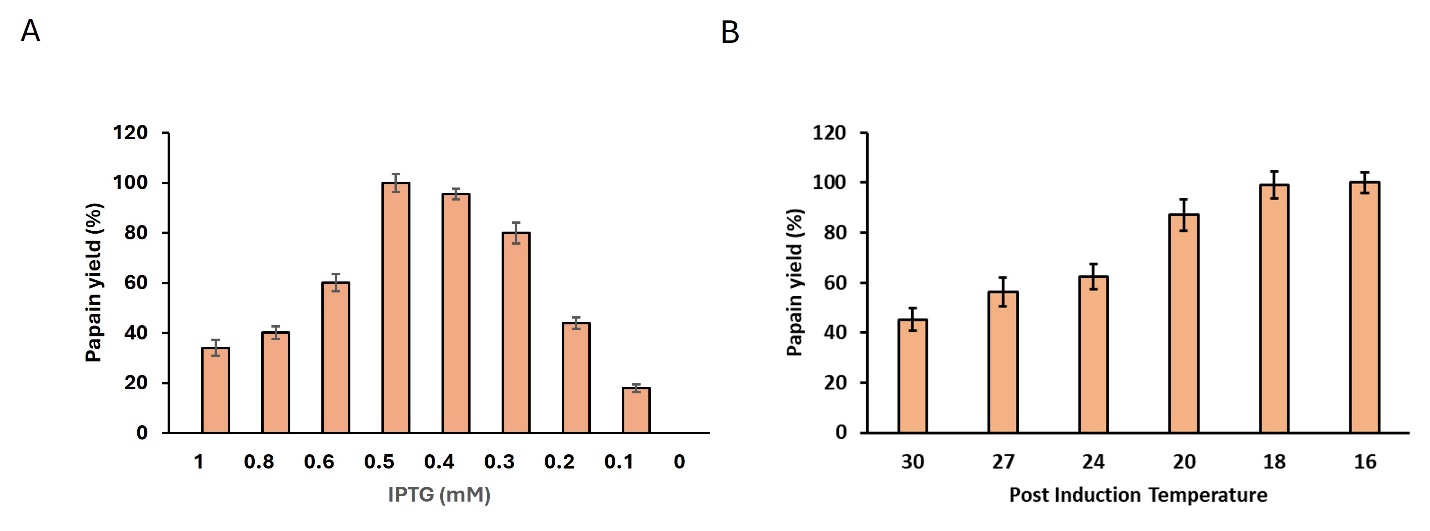


**FIG S5** Values of peptide yield as a function of: A) IPTG concentration and B) incubation temperature.


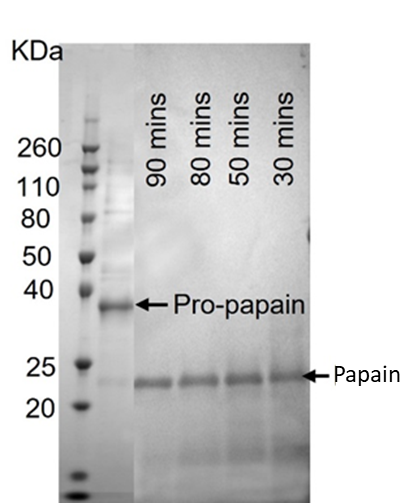


**FIG S6** A) Monitoring the extent of activation of pro-papain to mature papain by SDS-PAGE as a function of incubation time. Incubations were performed in the presence of 20 mM cysteine and 2 mM EDTA at 48 °C.

Enzyme 1. Pre-Propapain (Accession code: M15203.1) https://www.ncbi.nlm.nih.gov/nuccore/M15203.1


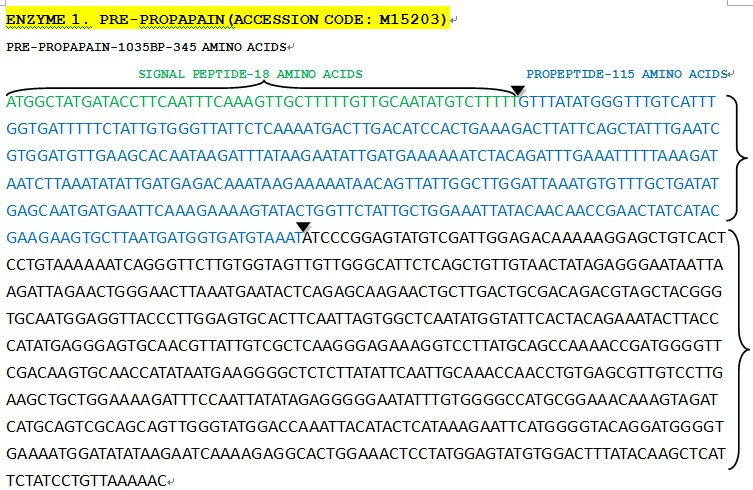


**FIG S7**. DNA sequence encoding the signal peptide (26 aa), pro-peptide (115 aa), and catalytic domain (212 aa).

| Protein names | Host cells | Cellular target | Yield | Solubility | References |
| --- | --- | --- | --- | --- | --- |
| Pro-papain | Spodoptera frugiperda Sf9 | Cytoplasm | ~300 µg/L | Soluble | (1) |
| Pro-papain | Saccharomyces cerevisiae | Cytoplasm | 1.7 mg/L | Soluble | (2) |
| Pro-papain | *Escherichia coli* | Cytoplasm | ~ 400 mg/L | Insoluble | (3) |
| Papain like protease (Triticain-α-GM) | *Escherichia coli* | Cytoplasm | 20-30 mg/L | Insoluble | (4) |
| Pro-papain | Engineered *Escherichia coli* | Cytoplasm | ~349 mg/L | Soluble | This study |

Table S1

Comparison of papain and papain like protease expression with different host cells.

References:

1. Vernet T et al,. Secretion of functional papain precursor from insect cells: Requirement for N-glycosylation of the pro-region. *Journal of Biological Chemistry (1990) 265(27)*
2. M K Ramjee et al,. A novel yeast expression/secretion system for the recombinant plant thiol endoprotease propapain. Protein Eng. 1996 Nov;9(11):1055-61.
3. Choudhury D, Roy S, Chakrabarti C, Biswas S, Dattagupta JK. 2009. Production and recovery of recombinant propapain with high yield. Phytochemistry 70.
4. Neonila V. Gorokhovets et al,. Rational Design of Recombinant Papain-Like Cysteine Protease: Optimal Domain Structure and Expression Conditions for Wheat-Derived Enzyme Triticain-α. *Int. J. Mol. Sci.* 2017, *18*, 1395
